# Altered neutrophil-to-lymphocyte ratio in sepsis secondary to canine parvoviral enteritis treated with and without an immunomodulator in puppies

**DOI:** 10.3389/fvets.2022.995443

**Published:** 2022-11-08

**Authors:** Adriana I. Muñoz, José Luis Maldonado-García, Ana Fragozo, Luis Vallejo-Castillo, Amellalli Lucas-Gonzalez, Ismael Trejo-Martínez, Lenin Pavón, Gilberto Pérez-Sánchez, Laura Cobos-Marin, Sonia Mayra Pérez-Tapia

**Affiliations:** ^1^Unidad de Desarrollo e Investigación en Bioterapéuticos (UDIBI), Escuela Nacional de Ciencias Biológicas, Instituto Politécnico Nacional, Mexico City, Mexico; ^2^Departamento de Inmunología, Escuela Nacional de Ciencias Biológicas, Instituto Politécnico Nacional, Mexico City, Mexico; ^3^Laboratorio de Psicoinmunología, Dirección de Investigaciones en Neurociencias, Instituto Nacional de Psiquiatría Ramón de la Fuente Muñiz, Mexico City, Mexico; ^4^Laboratorio Nacional para Servicios Especializados de Investigación, Desarrollo e Innovación (I + D + i) para Farmoquímicos y Biotecnológicos (LANSEIDI-FarBiotec-CONACyT), Mexico City, Mexico; ^5^Laboratorio de Virología, Facultad de Medicina Veterinaria y Zootecnia, Universidad Nacional Autónoma de Mexico, Mexico City, Mexico

**Keywords:** canine parvoviral enteritis, neutrophils, lymphocytes, cortisol, epinephrine, norepinephrine, human dialyzable leucocytes extract

## Abstract

Neutrophil-to-lymphocyte ratio (NLR) is a cheap and easy-to-obtain biomarker that mirrors the balance between innate and adaptive immunity. Cortisol and catecholamines have been identified as major drivers of NLR. High cortisol levels increase neutrophils while simultaneously decreasing lymphocyte counts. Likewise, endogenous catecholamines may cause leukocytosis and lymphopenia. Thus, NLR allows us to monitor patient severity in conditions such as sepsis. Twenty-six puppies with sepsis secondary to canine parvoviral enteritis were treated with and without an immunomodulator. Our group determined the NLR and the plasmatic cortisol levels by chemiluminescence, and norepinephrine (NE) and epinephrine (E) by HPLC during the first 72 h of clinical follow-up. Our results showed that at admission puppies presented an NLR value of 1.8, cortisol of 314.9 nmol/L, NE 3.7, and E 3.3 pmol/mL. Both treatments decreased admission NLR values after 24 h of treatment. However, only the puppies treated with the immunomodulator (I) remained without significant changes in NLR (0.7–1.4) compared to the CT group, and that showed a significant difference (*P* < 0.01) in their NLR value (0.4–4.6). In addition, we found significant differences in the slope values between the admission and final values of NLR (*P* < 0.005), cortisol (*P* < 0.02), and E (*P* < 0.05) between treatments. Then, our data suggest that the immunomodulator positively affects the number of lymphocytes and neutrophils involved in NLR as well as major drivers like cortisol and epinephrine, which is reflected in clinical parameters and survival.

## Introduction

Canine parvoviral enteritis (CPE) is caused by canine parvovirus type 2 (CPV-2), a member of the *Parvoviridae* family (1, 2). This viral disease mainly affects puppies, produces mucoid hemorrhagic gastroenteritis, and has been estimated to have a low survival rate of 9% with no treatment and 64% with treatment (3, 4) secondary to sepsis (2, 5). This is a consequence of the main clinical manifestations of CPE, destruction of the intestinal crypts, atrophy of the thymus, and aplasia of the bone marrow, which favor bacterial translocation, immunosuppression and neutropenia (6, 7). Further, dogs with CPE show high TNF-α, IL-1β, IL-6, C-reactive protein, ceruloplasmin, and ferritin concentrations; alterations in hemostasis and renal and hepatic function; and failure of other organs (2, 5, 8).

The neutrophil-to-lymphocyte ratio (NLR) is a cheap and easy-to-obtain biomarker that reflects the balance between innate and adaptive immune responses (9). Therefore, its elevation values should be observed in any pathology with systemic inflammatory response syndromes, such as bacterial or fungal infections, myocardial infarction, trauma, and post-surgical complications (10). In some conditions, elevated NLR has been associated with raised mortality in heart disease, influenza, pneumonia, and chronic kidney disease (11). Conversely, low NLR suggests an adequate balance in the inflammatory response and is often interpreted as a favorable prognostic factor (9). The NLR value is indirectly influenced by the circulating concentrations of cortisol and endogenous catecholamines, which are its major drivers (9, 10). Elevated blood levels of cortisol may cause neutrophilia and lymphopenia (12) while changes in blood levels of catecholamines, as epinephrine, may cause leukocytosis and lymphopenia (13). Despite this, the reports of clinical follow-ups that present NLR values commonly do not show the concentrations of catecholamines and cortisol.

In dogs, NLR has been recently reported to be helpful in the prognosis of puppies with acute diarrhea (14) and the diagnosis and prognosis of dogs with inflammatory bowel disease (15). Additionally, NLR and cortisol concentrations are the main prognostic markers of CPE (16, 17). In this study, our group evaluated changes in NLR, cortisol, and catecholamine in puppies with sepsis secondary to CPE. We compare two groups of puppies, one treated conventionally vs. another that received an immunomodulator as adjuvant treatment. The immunomodulator is composed of a complex mixture of low-molecular-weight peptides obtained from human buffy coat (18). The most abundant peptide of this mixture is extracellular monomeric ubiquitin (mUb) and a variant lacking both C-terminal glycine residues (UbΔGG) (18). Recently, we reported that the addition of this immunomodulator to CPV2- infected puppies significantly increases survival (19).

This manuscript aims to show that NLR, cortisol, and catecholamine levels are early and reliable parameters to predict the survival of CPV2-infected puppies with secondary CPE despite their breed, weight, size, and age.

## Materials and methods

Twenty-six CPV-2-positive puppies under 6 months of age that exhibited at least two clinical criteria for sepsis and required hospitalization participated in this study. The remaining inclusion, exclusion, and elimination criteria can be found in the Supplementary Table S1. The recruitment of participating puppies was performed in veterinary hospitals from March 2019 to February 2022, with permission from the Research Ethics Committee of the National School of Biological Sciences of the National Polytechnic Institute (ENCB-IPN) (ZOO-015-2019) and the informed consent of the owners. The stool test to confirm infection was end-point polymerase chain reaction (10).

Puppies were divided into two groups; the first received conventional treatment (CT), CT aims to minimize the discomfort characteristic of this clinical condition, limiting diarrhea, vomiting, dehydration, and opportunistic infections, and the second group additionally received the immunomodulator (I) as adjuvant treatment. The CT was established according to the clinical criteria of the veterinarian, the Supplementary Table S2 describes the CT used in each group. Pharma-FT from Mexico City, Mexico, donated the oral immunomodulator Transferon^®^ (batch number 18A03). The dose (0.5 mg/24 h) was administered subcutaneously for 5 days, while the conventional treatment was administered at the decision of the veterinarian, its average duration was 6 days.

In all cases, blood samples were obtained between 9 and 12 h from the jugular vein using microtainer with anticoagulant EDTA (BD Vacutainer^®^, New Jersey, USA) at admission and 24 (Time 1), 48 (Time 2), and 72 h (Time 3) post-hospitalization. The volume obtained was 1–3 mL. The period in which the evaluations were performed corresponds to the critical period of the disease, in which the highest percentage of mortality occurred. Blood samples were placed under refrigeration (5 ± 3 °C) until processing.

The number of neutrophils and lymphocytes was obtained from a peripheral blood smear in a differential leukocyte count. This data was used to get the absolute cell count, which was quantified through a semi-automated hematology analyzer CELL-DYN^®^ Emerald (Abbot, Illinois, USA) and electrical impedance; the equipment was verified and calibrated for the species.

The blood samples were centrifuged at 1,233 x g for 10 min, and the plasma obtained was stored at −20 °C. Quantification of plasma cortisol was performed in an Architect i2000SR immunoassay analyzer (Abbott, Illinois, USA), using a competitive heterogeneous chemiluminescent microparticle immunoassay. Epinephrine and norepinephrine concentrations were determined by high performance liquid chromatography (HPLC) as previously reported (19). Briefly, norepinephrine and epinephrine were extracted from 250 μl plasma by adding 250 μl extraction buffer containing 5% ascorbic acid, 200 mM sodium phosphate, 2.5 mM L-cysteine, 2.5 mM EDTA, and 2.4 M perchloric acid. The mixture was incubated at – 20 °C for 10 min. The supernatants containing norepinephrine and epinephrine were collected after centrifugation at 12,419 × *g* and 4 °C for 10 min. Samples were passed through 0.22 μm filters and they were processed by solid-phase extraction using a Hypersep C18 cartridge (Thermo Scientific, Massachusetts, USA). Neurotransmitter concentrations were identified by reversed-phase HPLC in a system consisting of a PU-2089 plus pump (Jasco Inc., Japan) and an X-LC™ 3120FP fluorescence detector (Jasco Inc., Japan). The instruments were controlled using ChromNav software (Jasco Inc., Japan). Chromatographic runs were carried out with a Jupiter C18 column (300 Å, 5 μ, 4.6 × 250 mm, Phenomenex^®^) at 30 °C. The column was in equilibrium with mobile phase A containing 0.1% trifluoroacetic acid in water. A linear gradient was run from min 5 to min 20 with mobile phase B containing 0.1% trifluoroacetic acid in acetonitrile. The flow rate was 0.8 mL/min. The fluorescence detector was set at a gain of 1,000, attenuation of 32, the response of 20 s, excitation at 280 nm and emission of 315 nm. The injection volume of the sample was 80–100 μL.

We calculated the median and confidence interval of the admission value and the values obtained at 24, 48, and 72 h in the CT and I of the number of lymphocytes, neutrophils, and the plasmatic concentration of cortisol, norepinephrine, and epinephrine. The NLR was calculated using the following formula:


$$\begin{array}{l} NLR=\frac{absolute\;number\;of\;neutrophils}{absolute\;number\;of\;lymphocytes} \end{array}$$

The values obtained were organized by time and treatment, and a Shapiro-Wilk normality test was performed, after which a Kruskal-Wallis test and Dunn's test as a *post-hoc* test were applied. The slope (*m*), generated by the changes in analyte levels, secondary to the treatments, was calculated with the values on days 1 and 3 using the formula:


$$\begin{array}{l} m=\frac{value\;of\;analyte\;at\;day\;3-value\;of\;analyte\;ad\;day\;1}{day\;3-day\;1} \end{array}$$

The values obtained were organized by analyte and treatment, and a Shapiro-Wilk normality test was performed, after which a student *t*-test was applied. The survival was calculated with Mantel-Cox test. Analytes are described with median and confidence interval at 95% or with mean in the case of slopes. A statistical analysis was performed using GraphPad Prism v9.4.0 (San Diego, California, USA). A statistical significance was considered when *P* < 0.05.

## Results

As expected, the puppies evaluated in the study presented a wide distribution in breed, weight, gender, and age (demographic data is shown in Supplementary material). This heterogeneity in the analyzed sample causes a wide dispersion of the data on lymphocyte and neutrophil numbers as well as plasma cortisol, epinephrine, and norepinephrine plasmatic levels (Table 1). Due to the wide dispersion of the data, no changes induced by the treatment were detected through a direct comparison between metabolite levels assessed. The predominant strain of CPV2 causing canine parvoviral enteritis in our study population was CPV-2c (88.5%), a small number of puppies presented the CPV-2b (11.5 %), some authors have mentioned that CPV-2c induces a more severe clinical presentation (20, 21).

**Table 1:** Values of neutrophils, lymphocytes, cortisol, and catecholamines in puppies with sepsis secondary to canine parvoviral enteritis at admission and along 72hrs of treatment.

	**Lymphocytes**	**Neutrophils**	**Cortisol**	**Epinephrine**	**Norepinephrine**
	**1x10^9^/L**	**1x10^9^/L**	**nmol/L**	**pmol/mL**	**pmol/ mL**
Admission	1.55 (0.70–3.00)	2.45 (0.30–11.60)	413.90 (91.80–634.60)	3.31 (0.00–11.10)	3.71 (0.40–15.80)
CT1	1.22 (0.70–10.20)	0.85 (0.00–6.50)	237.30 (82.80–678.70)	6.64 (4.27–8.86)	1.14 (0.12–4.06)
CT2	1.67 (1.00–4.00)	3.85 (0.20–9.00)	83.15 (22.10–233.00)	4.08 (0.00–9.07)	1.13 (0.00–2.55)
CT3	1.05 (0.90–3.50)	9.30 (2.80–17.70)	81.40 (11.30–198.60)	1.69 (0.00–6.53)	0.63 (0.00–5.07)
I1	2.50 (0.50–5.00)	2.05 (0.30–3.50)	124.20 (11.00–469.00)	1.76 (0.00–10.07)	5.76 (1.72–16.54)
I2	1.65 (0.20–7.80)	1.60 (0.00–17.30)	140.70 (11.00–664.90)	3.20 (0.00–15.45)	4.43 (1.42–6.00)
I3	3.25 (0.60–5.40)	4.20 (0.20–10.80)	59.30 (11.00–618.00)	2.34 (0.00–12.28)	2.93 (1.05–17.50)

The variables are described with the median and the confidence interval at 95%. The statistical analysis did not identify differences between the beginning of the values, the times evaluated, and the groups. CT = conventional treatment; I = immunomodulator.

**Figure 1:**
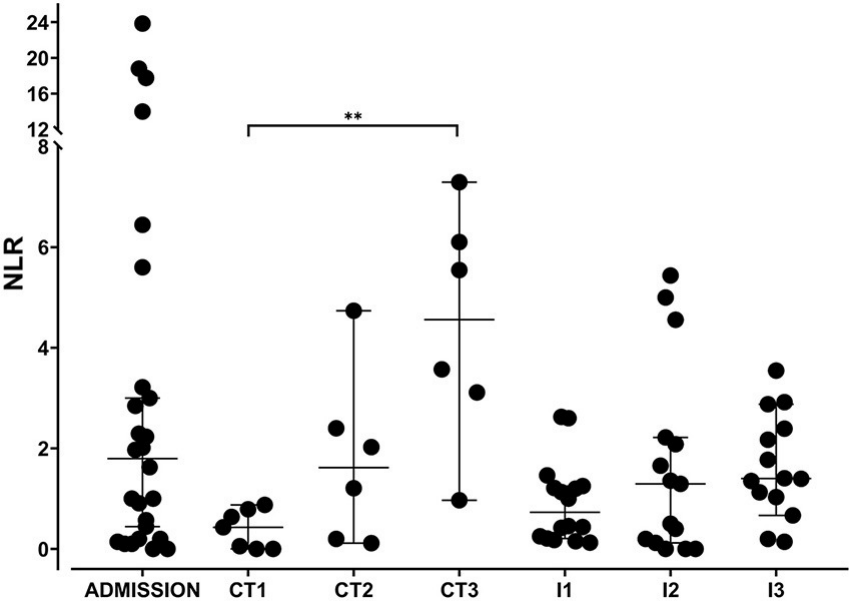
Neutrophil to lymphocyte ratio (NLR) in puppies with sepsis secondary to canine parvoviral enteritis. The NLR was obtained from the ratio of the absolute count of neutrophils and lymphocytes in puppies with sepsis secondary to canine parvoviral enteritis treated with conventional treatment and those that received an immunomodulator as an adjuvant treatment. The graph shows NLR at admission and three subsequent days of treatment (critical time). Each dot represents a puppy. There are statistical differences between days 1 and 3 in the conventional treatment group. Kruskal–Wallis test, Dunn's *post-hoc* test. Median ± 95% interval confidence. ** = *P* ≤ 0.01. CT = conventional treatment; I = immunomodulator.

The survival results between groups showed a significant difference (*P* < 0.0243, log-rank test) since puppies administered with the immunomodulator showed an increase in the survival rate (87.5%) in comparison with puppies that received CT (50%) at day 3 of clinical follow-up. The NLR allowed us to observe the changes induced by each treatment (Figure 1). The comparison of admission values vs. CT or I at 24 h (CT1 and I1) of treatment suggests a reduction in NLR. The NLR values in CT showed a significant increase in CT1 (24 h) and CT3 (72 h) (*P* < 0.0063; H = 16.55, df = 90), which is associated with a deterioration in the clinical condition of the puppy. A lower NLR value is associated with better clinical status and prognosis (14). The median of the NLR in group I showed lower I2 (48 h) and I3 (72 h) values compared to CT.

**Figure 2:**
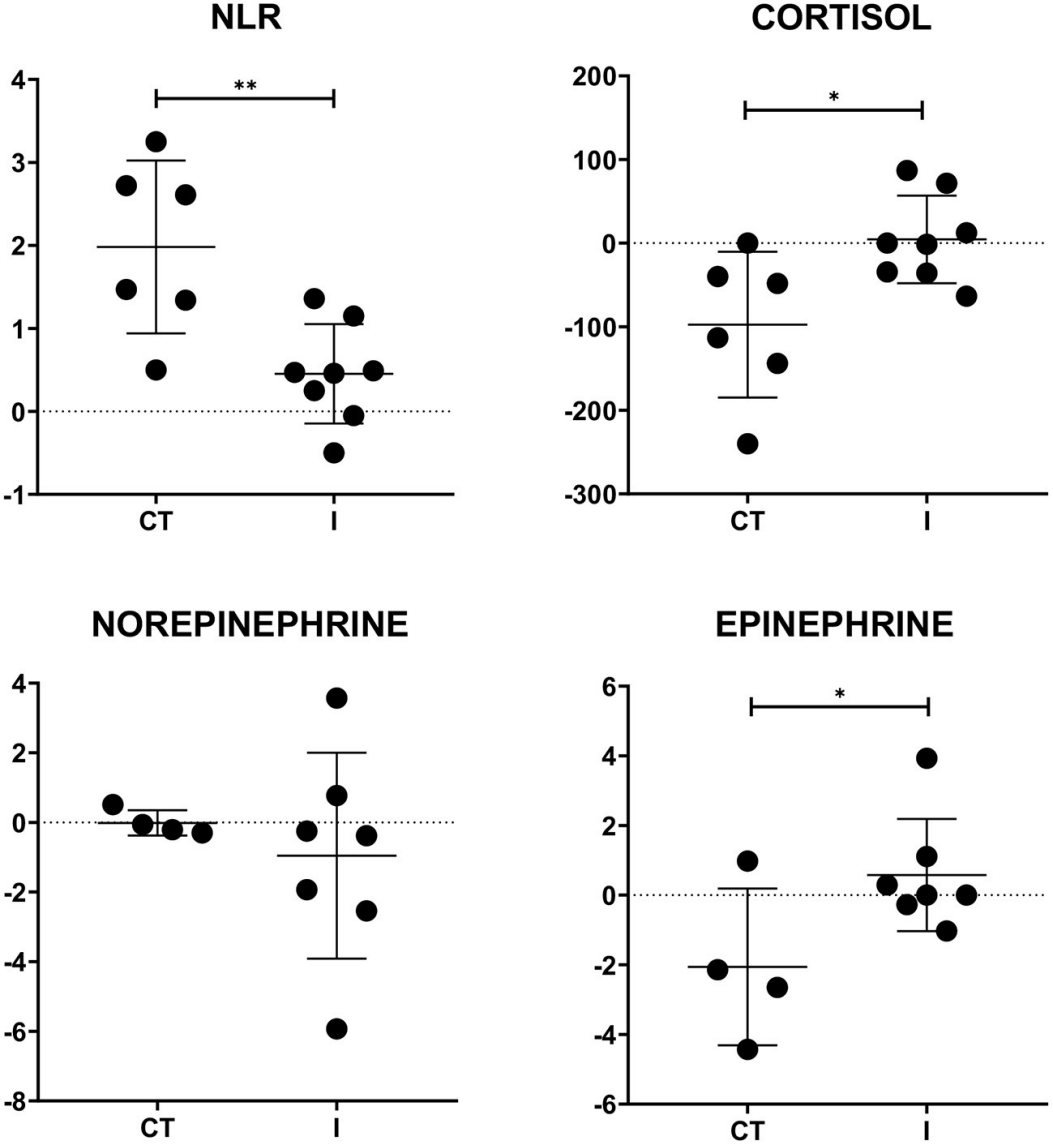
Slope values of NLR, cortisol, and catecholamines per treatment after 72 h. Slopes values of NLR and plasma cortisol were calculated per treatment using values of days 1 and 3. Statistical differences were observed in NLR, cortisol, and epinephrine slopes. A positive slope shows an increase in initial value while a negative one evidences a decrease. *t*-tests were carried out. Each dot represents a puppy. Mean ± SD. * = *P* ≤ 0.05; ** = *P* ≤ 0.01. CT = conventional treatment; I = immunomodulator.

The NLR values showed no statistical difference between treatments, possibly due to the wide dispersion of the data. To diminish the effect of data dispersion, the slopes of NLR, cortisol, and catecholamine levels induced by the treatments were calculated and compared in surviving dogs. The calculations were carried out during the critical period of the disease (1–3 days post hospitalization) in surviving dogs in which measurements of cortisol and catecholamines were made (Figure 2). Positive slopes show an increase while negative slopes show a decrease in comparison with initial values.

Our slope analysis evidenced differences induced by both treatments. Although both treatments caused an increase in NLR values, there was a significant difference (*P* < 0.0042; t = 3.480; *df* = 12) between the scores induced by the treatments. The complementary treatment using an immunomodulator (I) had a lower slope (NLR *m*_I_ = 0.45) than CT (NLR *m*_CT_ = 1.98). There was a significant difference in the cortisol slope between treatments (*P* < 0.0180; t = 2.737; *df* = 12). While the I group supplemented with an immunomodulator presented a light increase in the slope throughout the first 72 h (C *m*_I_ = 4.47), CT showed a significant decrease in its slope in the same period (C *m*_CT_ = −95.52). A similar pattern was observed in epinephrine (*P* < 0.0491; t = 2.274; *df* = 9) with sustained values for group I (E *m*_I_ = 0.58) and a decrease in CT (E *m*_CT_ = −2.06). Finally, both treatments showed similar behavior in the slopes corresponding to norepinephrine.

## Discussion

CPV-2 affects dogs of any breed, sex, and age, although a particularly affected population are puppies younger than 6 months. Its main complication is CPE, which is characterized by acute and severe gastroenteritis (1, 4). Sepsis is a common complication of CPE, and 82% of puppies with CPE are positive for circulating endotoxins (3). Hence, at admission, they show two or more criteria of systemic inflammatory response syndrome (SIRS) secondary to endotoxemia (3). Unfortunately, despite research and medical efforts, there is no consensus on the treatment and control of sepsis to date.

Consistent with state of the art, the puppies participating in this study also showed signs of SIRS. The high NLR values at admission are evidence of the impairment generated by the CPE since factors such as dehydration, vomiting, diarrhea, and infection contribute to the rise. The conventional treatment focuses mainly on rehydration, limiting vomiting, and antibiotics. It induces an evident decrease in NLR values, as observed in CT1 and I1 during the first 24 h of treatment.

Despite the initial improvement, the CT group presented a significant increase (*P* < 0.01) in NLR at 72 h; the puppies in this group showed a decrease in cortisol and epinephrine during the first 72 h after starting treatment. Although the decrease in circulating cortisol and epinephrine levels appeared positive, it was not reflected in the decrease in NLR values and a consequent improvement of the condition.

To understand this fact, we must take into account that increased circulating cortisol levels have diverse effects on immune response cells (12). *In vitro*, cortisol reduces mRNA levels of IL-1β, TNF-α, and IL-8, as well as IL-2 serum levels, causing lymphopenia during acute stress. Furthermore, cortisol causes an increase in the maturation of neutrophils in the bone marrow and their mobilization to the circulation in Wistar rats (20). Additionally, there is a decrease in NADPH oxidase p47phox subunit and enzymes like COX-2 and iNOS, which together decrease the effector capacity of neutrophils (22).

Although they are essential for tolerance against sepsis, endogenous glucocorticoids help to counteract excessive inflammation, vascular defects, and hypoglycemia (23). It has been described that elevated cortisol levels increase mortality in patients with sepsis (24). Therefore, a reduction in cortisol should have improved the clinical condition of the puppies. However, a plausible explanation for what was observed in puppies treated with CT is that during sepsis in neutrophils, there is a decrease in glucocorticoid receptor (GR) binding capacity of neutrophils. This prevents them from dying by glucocorticoid-induced apoptosis, which contributes to the persistence of multiorgan damage (25), evident in CT since the number of neutrophils increased substantially. It has also been reported that in septic shock, there is an increase in GR in T lymphocytes, promoting apoptosis with the subsequent decrease in T lymphocyte count (25). This would explain the rise in the value of the NLR in this group.

Catecholamines like epinephrine and norepinephrine regulate the inflammatory response associated with cortisol (26); both are considered the major drivers of the NLR (9). However, the impact of catecholamines on the immune system has been poorly studied in sepsis. To the best of our knowledge, this is the first study to present endogenous catecholamine levels during sepsis in puppies.

It has been reported that the use of exogenous epinephrine in the early stages of sepsis is associated with an improved prognosis (27, 28) since exogenous epinephrine acts as vasopressor to improve the hemodynamic status of the patient (27). Furthermore, it has been reported that the exogenous administration of norepinephrine in mice and humans deregulates the immune response and thus it may contribute to the development of sepsis-induced immunoparalysis (29).

Epinephrine has pro- and anti-inflammatory effects mediated by adrenergic receptors (AR) in leukocytes, through the activation of α1- and β1-AR, which potentiate IL-1β production. On the other hand, β2-AR has been shown to have anti-inflammatory effects (30, 31). Furthermore, it has been reported that epinephrine has dose-dependent effects on human leukocytes as, at low doses, it causes a decrease in CD11b and CD62L expression in neutrophils and monocytes, respectively. In addition, IL-8 levels increase and leukocyte diapedesis is promoted (13). Epinephrine is able to inhibit LPS-induced responses by decreasing IL-1β, IL-8, and MCP-1 production (13), relevant during sepsis.

The CT group did not present changes norepinephrine levels during the first 72 h of treatment, although an evident decrease in epinephrine levels was observed. There were no relevant changes in the number of lymphocytes. Still, we can assume that the lymphocyte capacity to regulate the immune response is limited during sepsis. given that the number of neutrophils continues to increase, affecting NLR observed in CT.

In contrast, the group that received the immunomodulator as adjuvant treatment did not show an increase in NLR values during the first 72 h of treatment. This finding was concordant with an improvement in clinical condition and the survival rate of puppies in the group. Additionally, at a molecular level, circulating cortisol levels were very similar to those detected on the first day of treatment, which represented a significant difference *(P* < 0.01) concerning the behavior of the CT group, which showed a decrease in the same period.

Epinephrine presented a trend similar to that of cortisol in circulating levels of I group. Our data suggest that the induced changes in cortisol and epinephrine levels improve the NLR compartment during the first 72 h of treatment in the group, favoring the better condition and survival of the puppies in the group.

The immunomodulator used in this work was Transferon^®^, a complex mixture of peptides, of which 136 are the extracellular most abundant. The major peptide components are mUb and UbΔGG. The extracellular mUb exerts other immunoregulatory and anti-inflammatory functions mediated by CXCR4 (32). For example, it inhibits TNF-α secretion in peripheral mononuclear cells stimulated with LPS (33) and increases the production of anti-inflammatory cytokines such as IL-4, IL-10, and IL-13 in a model of sterile lung inflammation (34).

The anti-inflammatory and immunoregulatory properties of extracellular mUb have been observed *in vitro* and *in vivo* when administered orally or parenterally (33–35). When Transferon was administered subcutaneously, extracellular mUb likely activated the CXCR4 receptor of leukocytes, modulating the inflammatory response (18).

Vallejo-Castillo and coworkers have suggested that extracellular mUb may stimulate vagal nerve endings through CXCR4, increasing acetylcholine (ACh) levels systemically and modulating the immune response (18). Interestingly, ACh is a neurotransmitter that downregulates the immune response through its interaction with α7nAchRs expressed by leukocytes (36). For example, ACh inhibits NF-κB translocation and the activation of both the JAK2-STAT3 pathway and the inflammasome in macrophages, suppressing proinflammatory cytokine production (37). The regulation of the immune response by ACh depends on the activation of the vagus nerve by pathogen- and damage-associated molecular patterns or cytokines (38, 39).

Our hypothesis is that extracellular mUb may increase the vagal tone in puppies with CPE through the interaction between mUb-CXCR4, this last expressed in the vagus nerve. There is evidence of increased Ach levels secondary to the pharmacological inhibition of acetylcholinesterase (AChE) in a mice model of pulmonary and systemic inflammation with a lipopolysaccharide. The administration of pyridostigmine reduced macrophage and lymphocyte counts and suppressed TNF, IL-1β, IL-6, and IFN-γ plasma levels without altering anti-inflammatory cytokine levels (40). Galantamine, another AChE inhibitor, decreases inflammation and has been shown to be effective in animal models of endotoxemia, inflammatory bowel disease, lupus (41–43), and metabolic syndrome in humans (44). On the other hand, it has been reported that patients with Chron's or irritable bowel disease show high vagal tone associated with a decrease in cortisol, epinephrine, and inflammatory cytokines levels, with the subsequent improvement of the clinical condition (45).

In this work, the immunomodulator induced a slight and smooth decrease in cortisol and epinephrine levels during the first 72 h. Unlike the abrupt reduction in TC, it may be associated with the improvement of NLR and the subsequent amelioration of the clinical condition and survival of the puppies.

## Limitations

To obtain the highest benefit from the data presented here, we must point out that the sample of this study is small and shows wide variability in breed, size, and age. Additionally, the time of admission in terms of the evolution of the symptoms was different between subjects. Furthermore, a size effect should be considered when using the results presented in this research, and in subsequent studies, the authors should consider a larger sample size due to high rate of mortality of this disease. In addition, it would also be desirable to evaluate proinflammatory cytokines in subsequent studies to achieve a broader and deeper analysis.

## Conclusion

Although more studies are necessary to obtain more information from our data, we propose that using a Ub-containing immunomodulator rich in extracellular mUb improves sepsis in puppies through a more physiological decrease in cortisol and epinephrine levels. This has a positive impact on the numbers of circulating neutrophils and lymphocytes, so the molecular and cellular changes induced by the immunomodulator were associated with an improvement in the puppies' condition.

## Data availability statement

The original contributions presented in the study are included in the article/Supplementary material, further inquiries for scientific/academic purposes can be directed to the corresponding authors.

## Ethics statement

The animal study was reviewed and approved by Committee of the National School of Biological Sciences of the National Polytechnic Institute. Written informed consent was obtained from the owners for the participation of their animals in this study.

## Author contributions

AIM, JLM-G, LC-M, LP, and SMP-T generated the research idea and developed the proposal. AIM, JLM-G, AF, and LP carried out the statistical analysis, analyzed data, edited, and organized the manuscript. GP-S quantified the neurotransmitters. LV-C, AL-G, and IT-M reviewed and edited the original draft. All authors have read and approved the manuscript for its publication.

## Funding

Part of this work was performed using the equipment and facilities of Laboratorio Nacional para Servicios Especializados de Investigación, Desarrollo e Innovación (I + D + i) para Farmoquímicos y Biotecnológicos (LANSEIDI-FarBiotec-CONACyT), which is part of Unidad de Desarrollo e Investigación en Bioprocesos (UDIBI)-IPN. Resources were obtained from UDIBI at IPN (FTU/P3/19/03) and the Virology Laboratory of FMVZ at UNAM and by the Instituto Nacional de Psiquiatría Ramón de la Fuente Muñiz Project: NC092318.0 and FORDECYT-PRONACES/552265/2020.

## Conflict of interest

Author SMP-T is involved in the development and commercialization of oral Transferon. The remaining authors declare that the research was conducted in the absence of any commercial or financial relationships that could be construed as a potential conflict of interest.

## Publisher's note

All claims expressed in this article are solely those of the authors and do not necessarily represent those of their affiliated organizations, or those of the publisher, the editors and the reviewers. Any product that may be evaluated in this article, or claim that may be made by its manufacturer, is not guaranteed or endorsed by the publisher.
