# Supplementary material for: Altered neutrophil-to-lymphocyte ratio in sepsis secondary to canine parvoviral enteritis treated with and without an immunomodulator in puppies
Source: Front Vet Sci. 2022 Nov 8;9:995443. doi: 10.3389/fvets.2022.995443 (PMC9679511; doi:10.3389/fvets.2022.995443)
Supplement: Supplementary file 1 [file Data_Sheet_1.pdf]

Supplementary Material

Table S1. Inclusion, exclusion, and elimination criteria.

Inclusion criteria	• Any breed and sex under six months of age<br>• The appearance of clinical signs within a period no longer than 48 hours before consultation or diagnosis<br>• Minimum weight of 1 Kg<br>• Two or more clinical criteria for systemic inflammatory response syndrome (hypothermia < 37.8 °C or fever > 39.4 °C, tachycardia >140 bpm, tachypnea >30 rpm, and leukopenia < 5.5 x 10^9/L or leukocytosis > 12.5 x 10^9/ L)<br>• Necessary hospitalization<br>• Owners willing to participate in the clinical trial with a signed informed consent letter
Exclusion criteria	• Hospitalization under the observation of another veterinarian outside recruiting veterinary clinics/hospitals<br>• Necessary application of another immunomodulator or granulocytic colony-stimulating factor based on clinical evidence and at the discretion of a veterinarian
Elimination criteria	• Any adverse effects to administration of immunomodulator<br>• Suspended administration of immunomodulator<br>• Death within 24 hours after hospitalization

Table S2. Conventional treatment of the study population. Treatments were classified according to their action mechanism. The number of puppies that received each of the treatments is specified.

Treatment classification	Conventional treatment + vehicle<br>n= 10		Conventional treatment + immunomodulator<br>n= 16	
Fluid therapy	0.9% NaCl solution	10/10	0.9% NaCl solution	16/16
Antibiotics	Ampicilin	4/10	Ampicilin	2/16
	Metronidazole	4/10	Metronidazole	10/16
	Enrofloxacin	2/10	Enrofloxacin	2/16
	Cephalexin	4/10	Clindamicyn	2/16
	Sulfa-trimethoprim	3/10	Sulfa-trimethoprim	4/16
Antiemetics	Maropitant citatre	7/10	Maropitant citrate	11/16
	Metoclopramide	4/10	Metoclopramide	5/16
	Ondansetron	2/10		
Antiespasmodics	Prifinial	3/10	Butylhioscine	6/16
Gastric mucosa protectors	Ranitidine	5/10	Ranitidine	8/16
	Omeprazole	7/10	Omeprazole	14/16
			Sucralfate	5/16
Analgesics	Buprenorphine	7/10	Buprenorphine	10/16
	Meloxicam	2/10	Lidocaine (infussion)	3/16
	Lidocaine (infussion)	1/10	Metamizole	3/16
Parenteral nutrition	0.5% Dextrose and aminolyte	8/10	0.5% Dextrose and aminolyte	10/16

Table S3. Demographic characteristics of the study population.

Group	Age		Sex		Breed	
	Months	# of puppies	Sex	# of puppies	Breed	# of puppies
Conventional treatment + vehicle	2	3	Female	1	Mixed	3
	3	3				
	4	2	Male	9	Pure breed	7
	5	1				
	6	1				
Conventional treatment + immunomodulator	2	3	Female	8	Mixed	7
	3	2				
	4	5	Male	8	Pure breed	9
	5	3				
	6	3				
